# A custom-made weight-drop impactor to produce consistent spinal cord injury outcomes in a rat model

**DOI:** 10.1515/tnsci-2022-0287

**Published:** 2023-05-26

**Authors:** Ali Jarragh, Ali Shuaib, Ghanim Al-Khaledi, Fatima Alotaibi, Sulaiman Al-Sabah, Willias Masocha

**Affiliations:** Department of Surgery, Faculty of Medicine, Kuwait University, Kuwait City, Kuwait; Department of Physiology, Faculty of Medicine, Biomedical Engineering Unit, Kuwait University, Kuwait City, Kuwait; Department of Pharmacology & Toxicology, Faculty of Pharmacy, Kuwait University, Kuwait City, Kuwait; Department of Pharmacology and Therapeutics, Faculty of Pharmacy, Kuwait University, Kuwait City, Kuwait

**Keywords:** spinal cord injury, rat model, spinal cord impactor, contusion model

## Abstract

**Objective:**

The main objective of this study is to design a custom-made weight-drop impactor device to produce a consistent spinal cord contusion model in rats in order to examine the efficacy of potential therapies for post-traumatic spinal cord injuries (SCIs).

**Methods:**

Adult female Sprague-Dawley rats (*n* = 24, 11 weeks old) were randomly divided equally into two groups: sham and injured. The consistent injury pattern was produced by a 10 g stainless steel rod dropped from a height of 30 mm to cause (0.75 mm) intended displacement to the dorsal surface of spinal cord. The neurological functional outcomes were assessed at different time intervals using the following standardized neurobehavioral tests: Basso, Beattie, and Bresnahan (BBB) scores, BBB open-field locomotion test, Louisville Swim Scale (LSS), and CatWalk gait analysis system.

**Results:**

Hind limb functional parameters between the two groups using BBB scores and LSS were significantly different (*p* < 0.05). There were significant differences (*p* < 0.05) between the SCI group and the sham group for the hind limb functional parameters using the CatWalk gait analysis.

**Conclusion:**

We developed an inexpensive custom-made SCI device that yields a precise adjustment of the height and displacement of the impact relative to the spinal cord surface.

## 1 Introduction

Approximately 300,000 Americans live with permanent disability due to acute spinal cord injury (SCI) [1]. The number of prevalent cases of SCI globally was almost 27 million in 2016 [2]. A further understanding of the neurological regeneration potential has proven that central nervous system regeneration can be feasible by modulating the secondary inflammatory response, scarring, and/or myelination, and this discovery contradicts the previous belief that recovery was near impossible [3,4]. Rodents have been the most used animal models of SCI in the literature owing to their ease of handling, close pathophysiological resemblance to humans, and the fact that they are more cost-effective than many other models [3,5].

Spinal cord contusion models are more relevant to clinical practice than transection and distraction models since contusion is the most common injury mechanism in humans [6,7,8,9]. The secondary inflammatory response to SCI can be used in translational research for testing different potential experimental therapies [4].

The New York University (NYU)/Multicenter Animal Spinal Cord Impactor Study (MASCIS) impactor uses a weight-drop technique to induce contusion SCI [10]. On the other hand, the Ohio State University impactor and the Infinite Horizon impactor are computer-controlled electromagnetic devices that have also been developed for rat [11,12]. However, these systems are costly and not available in all research centers in the developing world.

For individual laboratories or small animal research facilities, a manually operated spinal cord impactor, which is relatively inexpensive and easier to install and operate, might be a feasible alternative. In this study, we developed a new customized SCI device that allows a precise adjustment of the height and displacement of the impact relative to the surface of the spinal cord.

## 2 Materials and methods

### 2.1 Animals

Adult female Sprague-Dawley rats (*n* = 24, 11 weeks old, 200–240 g) were randomly divided into two groups: sham (*n* = 12) and injured (*n* = 12). Sham-operated rats underwent the same surgical procedures, including a laminectomy, as the SCI group, although they did not sustain an SCI.

### 2.2 Customized spinal cord impactor device

**Figure 1:**
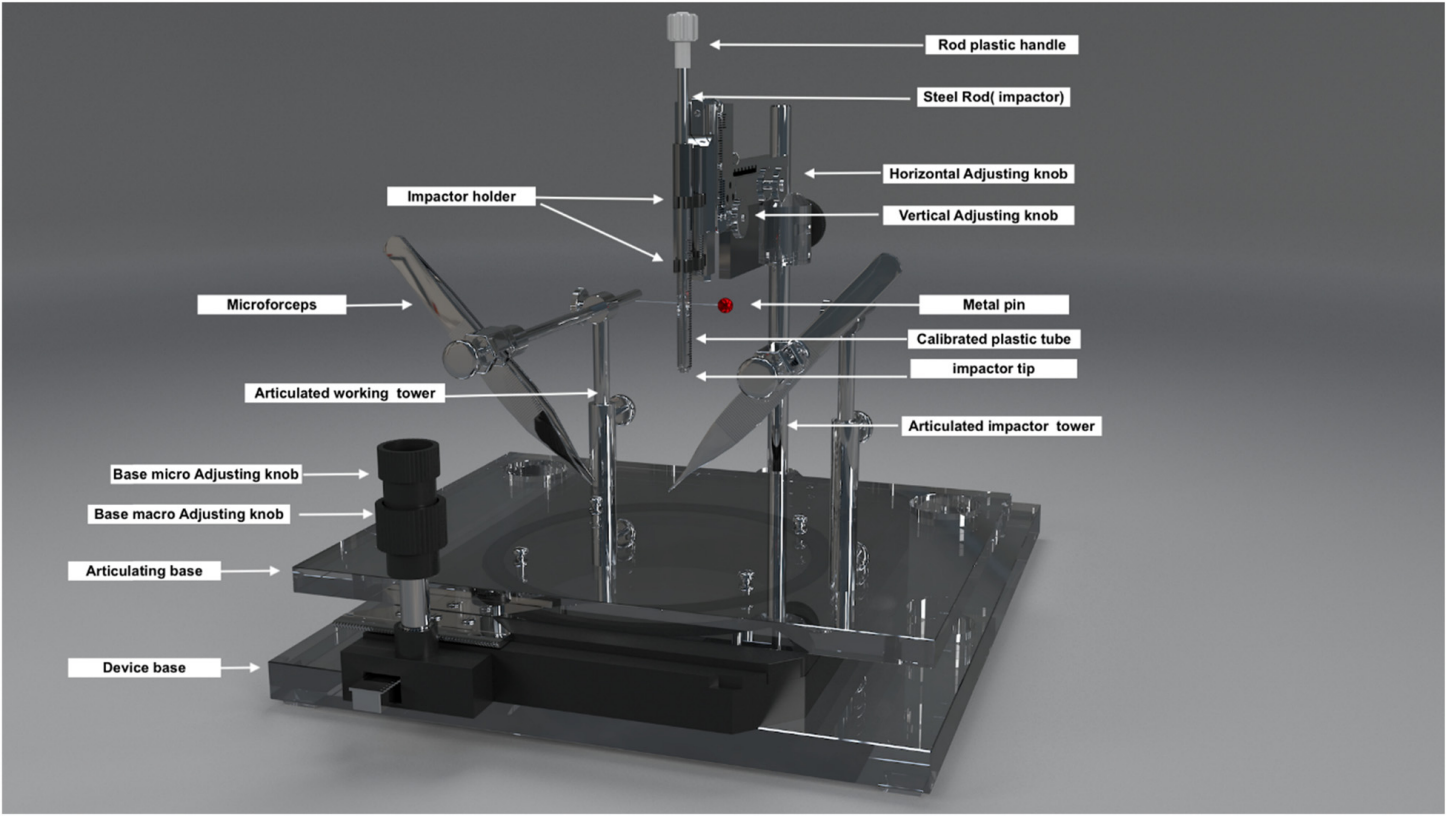
Customized weight-drop SCI impactor. A digitalized 3D model of the front view of the spinal cord impactor.

**Figure 2:**
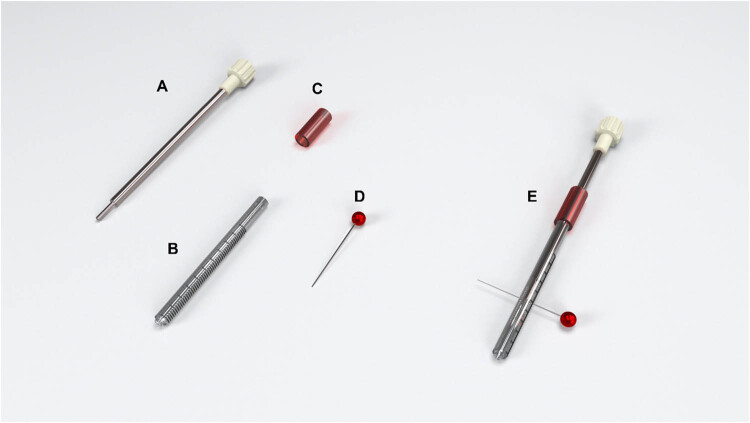
Impactor parts: (a) 10 g rod with a handle on top and a 2.0 mm impactor tip. (b) Plastic syringe. (c) The red tube is used to adjust the displacement of the rod into the spinal cord. (d) A metal pin is used for height adjustment. (e) The overall assembly of the impactor.

We used this customized impact device to induce experimental SCIs in rats by adopting Allen’s concept of dropping a known amount of weight from a known height to cause a contusive type of damage at the thoracic level (T10). The device consisted of an articulating base, two working towers, and an impactor dropping tower. The articulating base had two adjusting knobs for macro- and micro-motions in two planes to adjust the animal position under the impactor. The two working towers are made of stainless steel with an articulating connection that permits rotatory, horizontal, and vertical movements. The working towers can be used to attach the forceps and retractors to rigidly stabilize the spinal vertebrae. The impactor dropping tower has articulating parts that allow two planes of motion (Figure 1) (SDC: Video 1). The impactor part is made of a 10 g stainless steel rod, plastic tube, plastic stopper, and metal pin. We created the tube from a medical syringe with predrilled holes at regular intervals (25–35 mm) to adjust the height of the rod drop using a metal pin (Figure 2).

**Figure 3:**
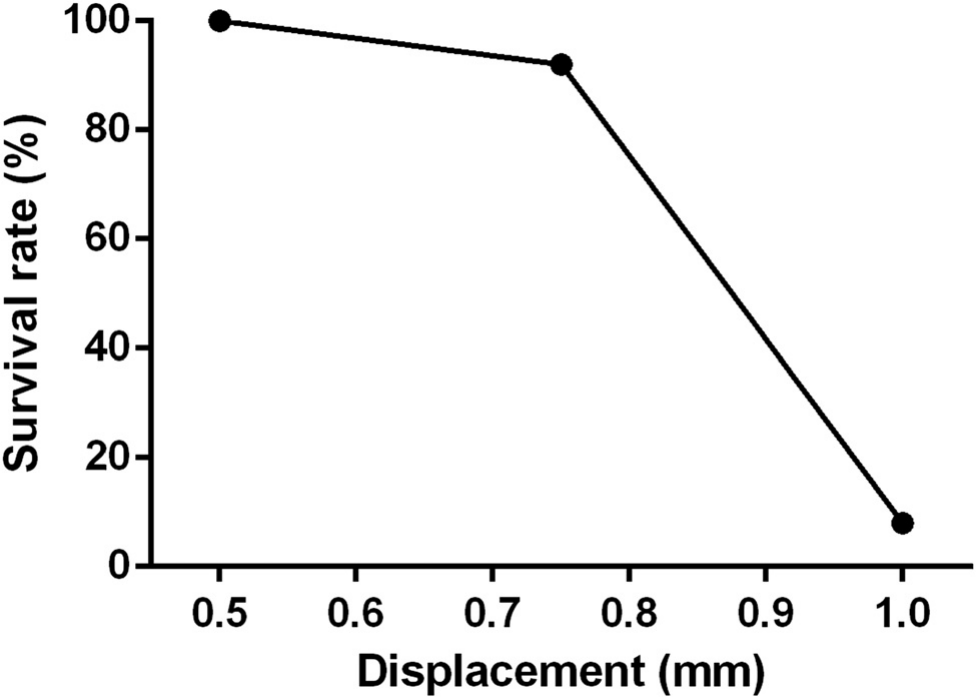
Survival rate 1-week post-SCI with 10 g weight free fall from 30 mm height with a depth of 0.5, 0.75, and 1.0 mm displacement of spinal cord dorsal surface.

The first part of this study aimed to standardize the surgical protocol and technique for the SCI device. To do this, the SCI device was tested in three groups of rats (*n* = 12 in each group) using three different displacement parameters (0.5, 0.75, and 1.0 mm) and a standard drop height of 30 mm (Figure 3). The results of this testing showed that the 0.5 mm displacement group resulted in a minimal and variable SCI effect, while the 1.0 mm displacement group had a high mortality rate. However, a consistent result with a moderate incomplete SCI injury was observed when the rod was dropped from a height of 30 mm and the displacement was set at 0.75 mm. Therefore, these parameters were chosen for use in this study.

**Figure 4:**
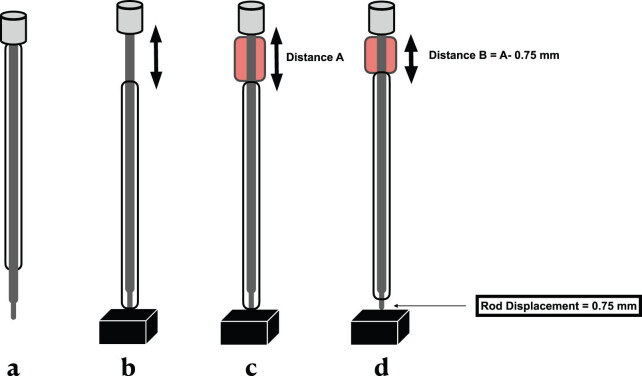
Displacement calibration. (a) The rod tube assembly. (b) The rod and tube are pressed against a hard flat surface. (c) A red plastic stopper is placed on top (Distance A = zero displacement). (d) Distance B was created by removing 0.75 mm from the red plastic tube (distance A) to create the desired displacement (0.75 mm).

**Figure 5:**
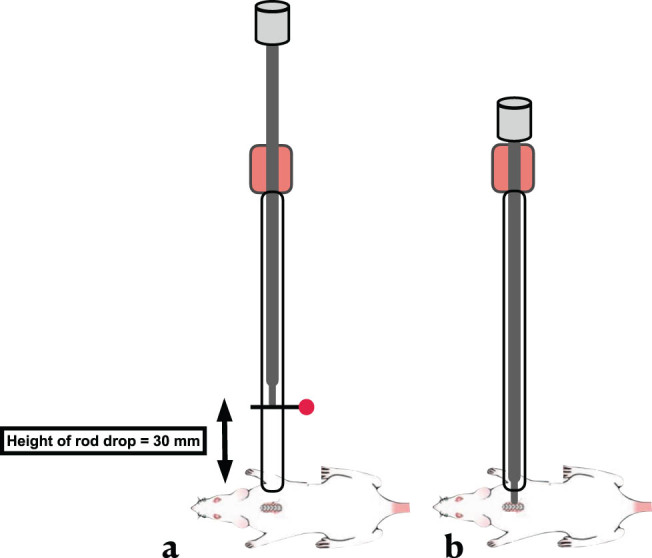
Height drop distance. (a) Height adjusted to 30 mm using a metal pin. (b) After releasing the pin, the rod causes spinal cord contusion at the T10 level.

The displacement calibration was conducted as follows: the tube rod assembly was pressed against a flat hard surface to make the tip flush to the same level as the lower edge of the tube, while the upper part of the rod with its handle was moved away from the upper part of the tube (Figure 4a and b). The distance between the upper rod handle and the upper part of the syringe was marked as the distance that caused zero displacement. A red plastic tube (stopper) was made with the same length of zero displacement distance (distance A: Figure 4c). The desired amount of displacement (distance B) was made by subtracting distance B from distance A, and in our case, 0.75 mm shortening was done to the red plastic tube (Figure 4d). The rod syringe assembly with its 0.75 mm distal protruded rod tip was lowered gradually until it touched the exposed dorsal surface of the spinal cord. After that, the impactor tip was lowered further to the intended displacement (0.75 mm) into the spinal cord and became flushed with the tube. This step was confirmed by compressed blood flow in the posterior spinal artery. Afterward, the rod was retracted, and the pin was placed at the desired height (30 mm). When we released the pin in the presence of the plastic stopper, the rod falls onto the spinal cord at a specific depth (Figure 5) (SDC 2: Video 2).

### 2.3 SCI surgery procedure

**Figure 6:**
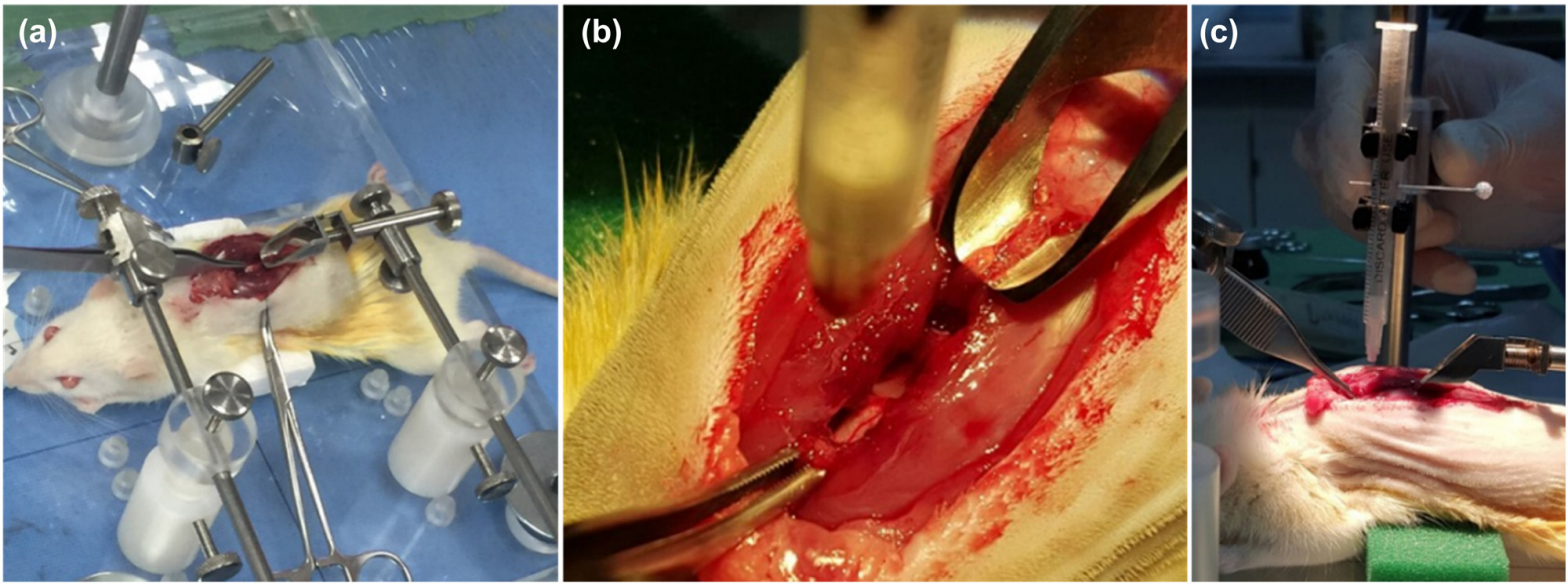
Surgical setup. (a) The spinous processes are stabilized with two holding forceps. (b) T10 laminectomy exposing the spinal cord and dorsal spinal artery is apparent. (c) Adjusting the impactor height perpendicular to the dorsa cord surface.

The animals were anesthetized with a mixture of 60 mg/kg ketamine HCl and 3.2 mg/kg xylazine intramuscularly. A 4 cm midline dorsal skin incision was made. A thoracic 10 (T10) laminectomy was performed to expose the spinal cord (Figure 6a and b); the intact dura matter was maintained, and pressure on the thecal sac was avoided. The spinous processes of the 9th and 11th vertebrae were rigidly clamped and fixed to the surgical frame to stabilize the spinal cord against displacement during injury (Figure 6a). The injury was inflicted by dropping a steel rod, 2 mm in diameter and weighing 10 g, from a height of 30 mm, while the tube was placed central and perpendicular to the exposed dorsal surface of the spinal cord (Figure 6c). There was no visible gross displacement of the spinal cord at the moment of impact. Subsequently, the lesion was verified by three researchers who identified the appearance of the hematoma on impact under a surgical microscope. Moreover, we observed hind limb twitching and tail lifting in rats, indicating successful spinal cord contusion with our custom-made impactor. In all experimental animals, this step was performed on all the rats using the same microsurgeon.

### 2.4 Neurological function test following SCI

An essential part of any experimental SCI study is the evaluation of the animals’ neurological statuses through neurological assessments during this study to provide direct evidence of whether a model has the potential to be used as an impactor [13]. For this objective, a battery of tests was incorporated in this research, including Basso, Beattie, and Bresnahan (BBB) scores [14], BBB open-field locomotion test, Louisville Swim Scale (LSS), and CatWalk gait analysis system (Noldus Information Technology, The Netherlands) [15,16]. All the analysis were carried out by two independent investigators who were blinded to the assigned groups.

### 2.5 Statistical analysis

All statistical analyses were performed using GraphPad Prism 7.0 software (GraphPad, La Jolla, CA, USA). The data were presented as mean  ±  SEM. The statistical differences between the two groups were analyzed using an established *t*-test, Kruskal–Wallis test followed by Dunnett’s multiple comparison test and two-way repeated-measures ANOVA followed by Bonferroni’s multiple comparison test. The difference was considered statistically significant at *p* < 0.05.


**Ethical approval:** The research related to animals’ use has been complied with all the relevant national regulations and institutional policies for the care and use of animals. All procedures were performed according to the protocol approved (approval code: ZM05/15) by the Animal Care and Ethics Committee of the Health Sciences Center, Kuwait University.

## 3 Results

**Figure 7:**
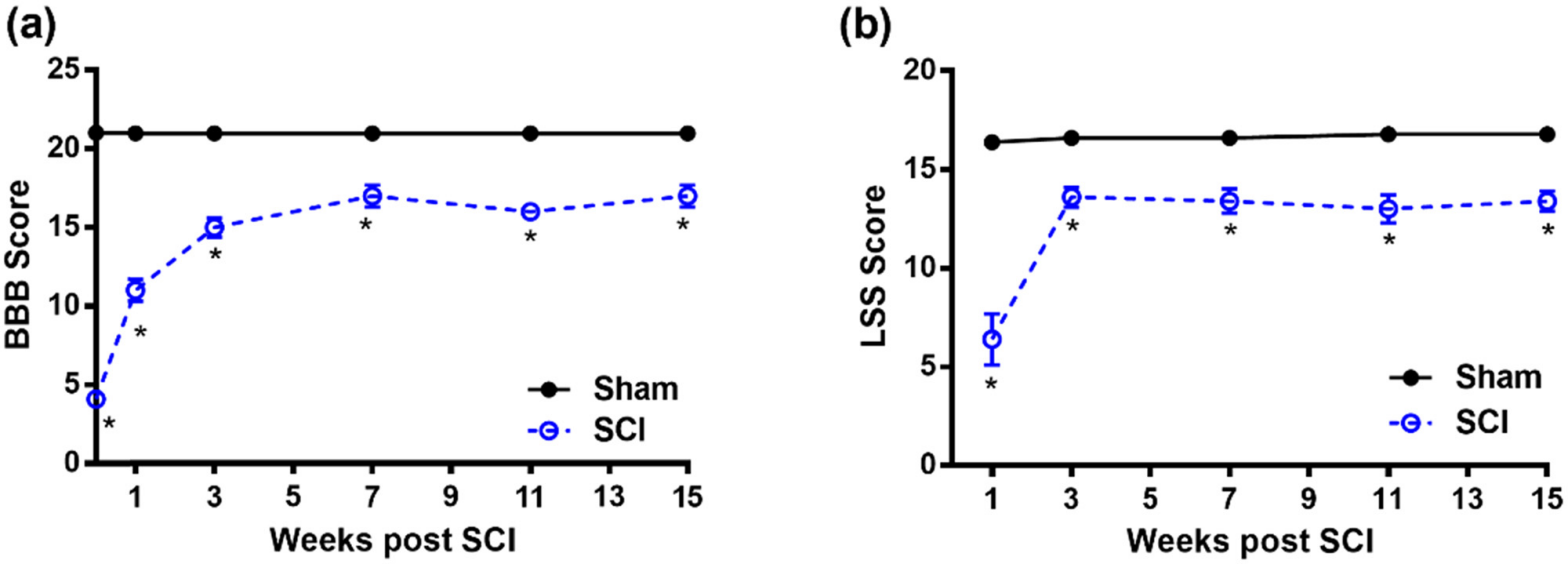
Recovery of hindlimb function: (a) the BBB scale and (b) the LSS score. Data are expressed as mean ± SEM. Statistically significant differences in comparison with sham-operated rats *p < 0.05 (two-way repeated-measures ANOVA followed by Bonferroni’s multiple comparison test).

The progress of the recovery of hindlimb function after incomplete moderate SCI was evaluated with the BBB scale in the sham-operated group (sham) and the SCI group on days 3, 7, 14, 21, 28, 35, 42, 49, and 56 after surgery (Figure 7a). There was a remarkable improvement in the BBB score of the SCI group within the first 14 days. For the injured group, the BBB curves appeared to be approaching a plateau at 5 weeks like what has been reported in other injury models [14,17]. Furthermore, the injury group’s BBB scores were significantly different from those of the sham group (*p* < 0.05).

Figure 7b shows the progress in the recovery of hindlimb function after incomplete moderate SCI as evaluated with the LSS in the sham-operated (sham) and SCI groups at weeks 1, 3, 7, 11, and 15. The LSS curves for the injured group appeared to be approaching a plateau at 3 weeks. Furthermore, the LSS scores for the injury group differed significantly from those of the control group (*p* < 0.05). It is important to note that at all time points where the BBB scores and LSS scores were evaluated, and the SCI group was compared to the sham group, the *p*-value was less than 0.0001.

**Figure 8:**
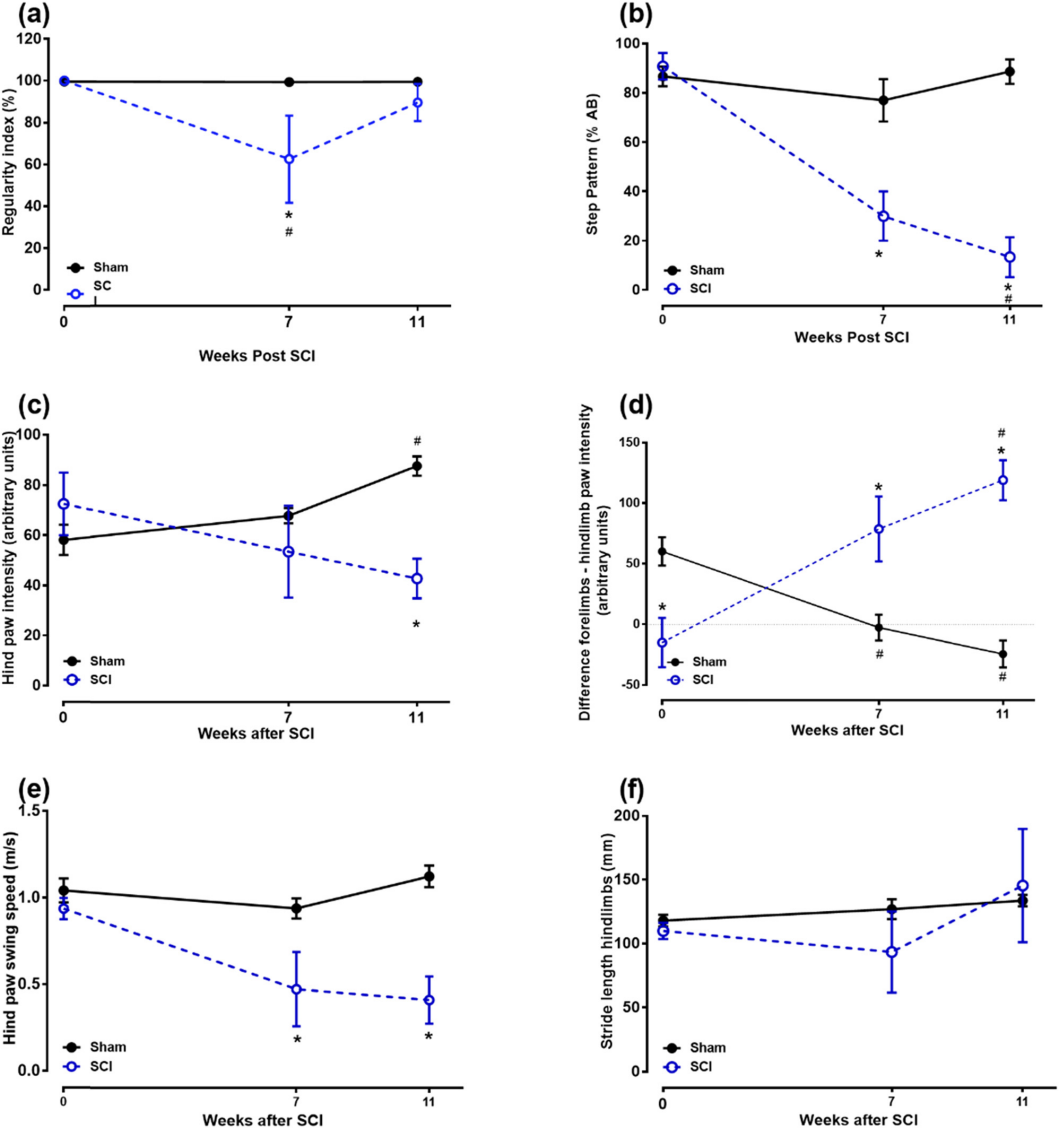
Paw parameters at week 0 and after SCI (weeks 7 and 11): (a) regularity index, (b) step pattern, (c) hind paw intensity, (d) forelimbs and hindlimbs paw intensity difference, (e) swing speed, and (f) hind paw stride length. Statistically significant differences in comparison with baseline: #*p* < 0.05 (Kruskal–Wallis test followed by Dunnett’s multiple comparison test). Statistically significant differences in comparison with sham-operated rats *p < 0.05 (two-way repeated-measures ANOVA followed by Bonferroni’s multiple comparison test).

Figure 8 shows the CatWalk gait analysis for the sham and SCI groups at weeks 0, 7, and 11. The interlimb coordination of the rats, measured by the regularity index, was 100% (Figure 8a) at baseline (week 0), indicating that the placements of the paws of all the rats followed a normal step sequence, and each paw was placed four times as the rat walked [15]. The interlimb coordination of the sham-operated rats did not change during the experiment (*p* > 0.05; Figure 8a). However, the regularity index of SCI rats decreased significantly to 62.5 ± 20.8% in week 7 post-operation compared to baseline and sham-operated rats (*p* < 0.05), indicating that SCI rats had lost interlimb coordination but recovered in week 11 (*p* > 0.05; Figure 8a).

Rats use the Ab alternate step sequence most of the time, and it decreases in SCI rats due to missteps [15]. The percentage of the Ab alternate step sequences from sham-operated rats did not change significantly during the course of the experiment (*p* > 0.05; Figure 8b). However, the percentage of the Ab alternate step sequence of SCI rats decreased significantly from 90.8 ± 5.3 to 30.0 ± 10.0% and 13.3 ± 8.2% in weeks 7 and 11 post-operatively, respectively (whole duration of the experiment) compared to baseline and sham-operated rats (*p* < 0.05; Figure 8b).

The pressure of the hind paw, which was measured using the light intensity of the hind paw [15] and represents the force used by the paws, increased over time for the sham-operated rats, while it decreased for the SCI rats (Figure 8c), and the difference between the two groups was significant at 11 weeks post-operation (*p* < 0.05; Figure 7c). SCI rats shifted weight/pressure from hindlimbs to forelimbs; therefore, the difference between forelimb intensity and hindlimb intensity increased significantly in weeks 7 and 11 post-operation (whole duration of the experiment) compared to baseline and sham-operated rats (*p* < 0.05; Figure 8d).

Swing speed reflects the velocity of the moving limb during the swing phase, and it has been reported to decrease in animals with SCI [18]. The swing speed of the sham-operated rats did not change significantly during the course of the experiment (*p* > 0.05; Figure 7e). However, the swing speed of SCI rats decreased significantly from 0.9 ± 0.1 to 0.5 ± 0.2 m/s and 0.4 ± 0.1 m/s in weeks 7 and 11 post-operation (whole duration of the experiment) compared to baseline and sham-operated rats (*p* < 0.05; Figure 8e).

There were no significant differences in hindlimb stride lengths between sham-operated and SCI rats compared to the baseline or between the two groups (*p* > 0.05; Figure 8f), which is like what has been reported in previous studies [15,19].

## 4 Discussion

The standardization of injury in SCI models can be accomplished by standardizing the animal physiological parameters and biomechanical parameters of the impactor device. The standardized physiological parameters in our experiment were the sex (females), body weight (200–240 g), and age (77 days) of Sprague-Dawley rats’ species. Studies have shown that the ideal age for a rodent animal model is 77 days, in which the spinal cord diameter will be similar between males and females regardless of their body weight [20]. Most spinal cord contusion devices control biomechanical factors such as the impactor weight, force, speed, duration, and height of drop to produce different grades of injury severity [10,11,12]. The spinal cord can deform up to 1/3 of its volume without any axonal damage provided the deformation is gradual [21]. However, if the impact velocity exceeds the critical velocity (0.5–1.0 m/s), the site of injury impact will travel caudal and cephalic along the large myelinated axon, causing irreversible shearing injury to the axons [3]. The current spinal cord impactors can adjust the force and drop height to achieve the desired critical velocity value [22]. In this study, we describe a novel low-cost apparatus for SCI in a rat model. This spinal cord impactor device provides an effective method by which SCIs can be induced in laboratory animals such as rats. This spinal cord impactor allows investigators to standardize the biomechanical variables of SCIs in rats. The main advantages of this system are (1) easy handling, (2) reproducible impacts, (3) adjustable impactor falling height, and (4) adjustable displacement in the spinal cord. Thus, the functionality and versatility of the spinal cord impactor device can be readily adapted to various levels of SCI severity. There are many issues with drop-test devices from a biomechanical perspective, primarily the interaction between drop height and impact velocity. However, the critical velocity can be estimated in our model using a mathematical equation ( {V}_{f}^{2}] = {V}_{i}^{2}] + 2*a* \triangle Y] ) based on the facts that earth gravitational acceleration (*a*) is constant and the initial velocity ( {V}_{i}^{2}] ) equals zero (***V*** indicates the velocity in m/s, *a* indicates the Earth’s gravitational acceleration = 9.8 m/s^2^ \triangle Y] (drop height) in meter (m)). The calculated velocity was 0.77 m/s (( {V}_{f}] = \sqrt{0\left+(2\times 9.81\left\times 0.03)}] = 0.77 m/s) for a 10 g rod falling from a 30 mm height, which is within the acceptable range of the defined critical velocity (0.5 – 1.0 m/s) [3].

One of the main aims in this study was to obtain a reproducible injury, which was confirmed by the behavior tests (BBB, LSS, and CatWalk) and compared to the sham group. This study used a customized impactor device to establish the SCI model, which can be used to demonstrate the effectiveness of preclinical therapy. The BBB scores and the changes in CatWalk gait parameters such as step pattern and the regularity index of coordination during walking, print area, and swing speed were similar to what has been obtained by other groups using commercially available impactors such as the NYU impactor or MASCIS weight-drop device [23,24,25]. The locomotion assessment with gait analysis and the recovery of walking function has more clinical importance to the patient and the physicians than other parameters [26]. This customized model, although not proven to be superior to any commercially available SCI impactor device, can still be viewed as a viable option. By using this model, scientists and medical professionals can conduct their research with more confidence in the consistency of their results. This makes it an attractive option for them, especially when resources and funds are limited. A recent investigation showcased the development of another straightforward, custom-made weight-drop impactor device, emphasizing the growing necessity for creating SCI models to facilitate neurotherapeutic research in developing countries [27]. This trend underscores the importance of accessible and affordable tools in advancing scientific knowledge and therapeutic interventions for SCIs.

The main limitation of this study is that our device was not compared to the current gold standard devices on the market. Unlike commonly used devices, the finer details of the impact, such as the moment of contact, the velocity of impactor, and the spinal cord secondary motion, cannot be measured and recorded. In addition, there was no histopathological assessment of the injured spinal cord. This study focused on the most standardized recovery outcome, which was the locomotion assessment even though the recovery of sensory and autonomic reflex components could not be assessed.

## 5 Conclusion

We designed, built, and tested a unique and inexpensive spinal cord impactor for rat models. Furthermore, behavior tests were used to validate the patency of the apparatus. Our customized spinal cord impactor device provides an effective method for producing SCIs in a simple laboratory setting with minimal resources. The overall expense of our spinal cord impactor is subject to variation across different countries, primarily due to fluctuations in manufacturing costs and material prices. Nonetheless, the apparatus remains a more economical alternative to existing commercial devices, thereby presenting a viable option for researchers in the field of SCI studies.

## Supplementary Material

Supplementary video 1

Supplementary video 2
